# Strategies to implement SARS-CoV-2 point-of-care testing into primary care settings: a qualitative secondary analysis guided by the Behaviour Change Wheel

**DOI:** 10.1186/s43058-021-00242-6

**Published:** 2021-12-18

**Authors:** Patrick Kierkegaard, Timothy Hicks, A. Joy Allen, Yaling Yang, Gail Hayward, Margaret Glogowska, Brian D. Nicholson, Peter Buckle, on behalf of the CONDOR Steering Committee, Julian Braybrook, Paul Dark, Kerrie Davis, Eloise Cook, Adam Gordon, Anna Halstead, Dan Lasserson, Andrew Lewington, Rafael Perera-Salazar, John Simpson, Philip Turner, Graham Prestwich, Charles Reynard, Beverley Riley, Valerie Tate, Mark Wilcox

**Affiliations:** 1 NIHR London In Vitro Diagnostics Co-operative, Department of Surgery and Cancer, Imperial College London, St Mary’s Hospital, Praed Street, London, W2 1NY UK; 2 CRUK Convergence Science Center, Institute for Cancer Research & Imperial College London, Roderic Hill Building, South Kensington Campus, Exhibition Road, London, SW7 2AZ UK; 3 NIHR Newcastle In Vitro Diagnostics Co-Operative, Newcastle upon Tyne Hospitals NHS Foundation Trust, Newcastle upon Tyne, NE7 7DN UK; 4 Translational and Clinical Research Institute, The Medical School, Newcastle University, Newcastle upon Tyne, NE1 7RU UK; 5 Nuffield Department of Primary Care Health Sciences, University of Oxford, Radcliffe Primary Care Building, Radcliffe Observatory Quarter, Woodstock Road, Oxford, OX2 6GG UK; 6 NIHR Community Healthcare MedTech and In-Vitro Diagnostics Co-operative, Nuffield Department of Primary Care Health Sciences, University of Oxford, Radcliffe Primary Care Building, Radcliffe Observatory Quarter, Woodstock Road, Oxford, OX2 6GG UK

**Keywords:** SARS-CoV-2, COVID-19, Point-of-care testing, Primary care, Theoretical Domains Framework, Behaviour Change Wheel, Behaviour change technique taxonomy

## Abstract

**Background:**

The purpose of this study is to develop a theory-driven understanding of the barriers and facilitators underpinning physicians’ attitudes and capabilities to implementing SARS-CoV-2 point-of-care (POC) testing into primary care practices.

**Methods:**

We used a secondary qualitative analysis approach to re-analyse data from a qualitative, interview study of 22 primary care physicians from 21 primary care practices across three regions in England. We followed the three-step method based on the Behaviour Change Wheel to identify the barriers to implementing SARS-CoV-2 POC testing and identified strategies to address these challenges.

**Results:**

Several factors underpinned primary care physicians’ attitudes and capabilities to implement SARS-CoV-2 POC testing into practice. First, limited knowledge of the SARS-CoV-2 POC testing landscape and a demanding workload affected physicians’ willingness to use the tests. Second, there was scepticism about the insufficient evidence pertaining to the clinical efficacy and utility of POC tests, which affected physicians’ confidence in the accuracy of tests. Third, physicians would adopt POC tests if they were prescribed and recommended by authorities. Fourth, physicians required professional education and training to increase their confidence in using POC tests but also suggested that healthcare assistants should administer the tests. Fifth, physicians expressed concerns about their limited workload capacity and that extra resources are needed to accommodate any anticipated changes. Sixth, information sharing across practices shaped perceptions of POC tests and the quality of information influenced physician perceptions. Seventh, financial incentives could motivate physicians and were also needed to cover the associated costs of testing. Eighth, physicians were worried that society will view primary care as an alternative to community testing centres, which would change perceptions around their professional identity. Ninth, physicians’ perception of assurance/risk influenced their willingness to use POC testing if it could help identify infectious individuals, but they were also concerned about the risk of occupational exposure and potentially losing staff members who would need to self-isolate.

**Conclusions:**

Improving primary care physicians’ knowledgebase of SARS-CoV-2 POC tests, introducing policies to embed testing into practice, and providing resources to meet the anticipated demands of testing are critical to implementing testing into practice.

Contributions to the literature

- This is the first study that utilised an implementation science framework to examine primary care physicians’ attitudes and capability to implement SARS-CoV-2 point-of-care testing into routine practice.
- By using the Behaviour Change Wheel, this study systematically identified opportunities to address primary care physicians’ knowledge gaps around SARS-CoV-2 POC testing and the specific resources needed to meet the anticipated demands of testing.
- Using secondary qualitative analysis in connection with implementation science can generate new knowledge and reveal additional context without the cost of additional data collection to address new and important implementation questions.

## Background

The unprecedented disruptions of the SARS-CoV-2 pandemic have forced a paradigm shift in the way primary care operates, with several core functions being reorganised to facilitate remote-first care services with face-to-face consultations only being offered if considered necessary [1, 2]. Although these changes created new opportunities for patients to quickly and conveniently access care [3–5], evidence has shown that remote consulting can lead to diminishing personal connectedness between physicians and patients, loss of ability to perform targeted physical examinations, and an increase in workload pressures for physicians [6–9]. Moreover, some patient groups may not have access to, or the ability to use, appropriate technology to participate in remote consultations [10, 11]. Revising national guidance to encourage the increase of face-to-face appointments may help address these challenges but will require a multifaceted approach to minimise the risk of contagion whilst vaccine programmes continue to be rolled out.

Considering this, point-of-care (POC) tests for SARS-CoV-2 can play an instrumental role in enabling more face-to-face consultations as the disease enters a more endemic phase. POC tests for SARS-CoV-2 can help provide real-time and on-site detection of SARS-CoV-2 infection without the need for specialised laboratory equipment [12, 13]. Primary care physicians (PCPs) can increase the volume of face-to-face clinical encounters and use POC tests during (or very close to) the time of consultation to detect and prevent contagion within the clinic [14]. Additionally, POC tests can act as a safety measure to control and contain risk in view of the uncertainties concerning the efficacy of different vaccines [15], new variants of concern [16, 17], and complexities of vaccine hesitancy [18, 19].

Evidence relating to the implementation of POC testing in primary care settings is limited. Much of the work on SARS-CoV-2 POC tests has focused on modelling clinical and economic impact of those tests [20], which limits the generalisability of findings to real-world settings [21]. More work is needed to examine the complex processes required to successfully and sustainably implement POC testing into primary care practices as the introduction of a new testing regime can change work environments, introduce deviations from routine behaviours, and alter roles and responsibilities [22–24]. A potential means for addressing these questions is to use a structured approach to identify current gaps in implementation strategies for embedding SARS-CoV-2 POC testing into everyday practice based on input from PCPs. This could, in turn, aid in the development of optimal strategies to facilitate the uptake and integration of SARS-CoV-2 POC testing among primary care practices.

To address this, we used implementation science, defined as ‘the scientific study of methods to promote the systematic uptake of research findings and other evidence-based practices into routine practice’ [22], to identify the factors affecting the implementation of SARS-CoV-2 POC testing to ensure it is optimally integrated into primary care [25, 26]. We opted to use an implementation science framework to theoretically guide the systematic identification of barriers to implementation and inform the development of strategies to facilitate the integration of SARS-CoV-2 POC testing into clinical practice. We chose a theory-based approach as it can facilitate a better understanding of the generalisability and replicability of our findings [27] and is recommended by the UK Medical Research Council (MRC) guidelines for developing and evaluating interventions as a means to increase intervention effectiveness [28].

We therefore used the Behaviour Change Wheel (BCW) [29], a multiphase process guide for developing complex behaviour change interventions, to examine and address the challenges of implementing POC testing into primary care. We drew from the associated Theoretical Domains Framework (TDF) [30], and Behaviour Change Techniques Taxonomy (BCTTv1) [31], to strengthen the link between theory, targeting interventions, and implementation planning [32]. We opted to use these frameworks and models because they provide a comprehensive theoretical coverage that integrates constructs from multiple behaviour theories. In addition, they are linked to evidence-based intervention functions that can assist with the translation of theory-informed intervention strategies into practice. They are interlinked, and using them together can help systematically guide the process of diagnosing challenges/enablers to implementation and identifying the intervention mechanisms that are likely to support the implementation of SARS-CoV-2 POC testing. We describe this process in more detail in the text below.

**Fig. 1:**
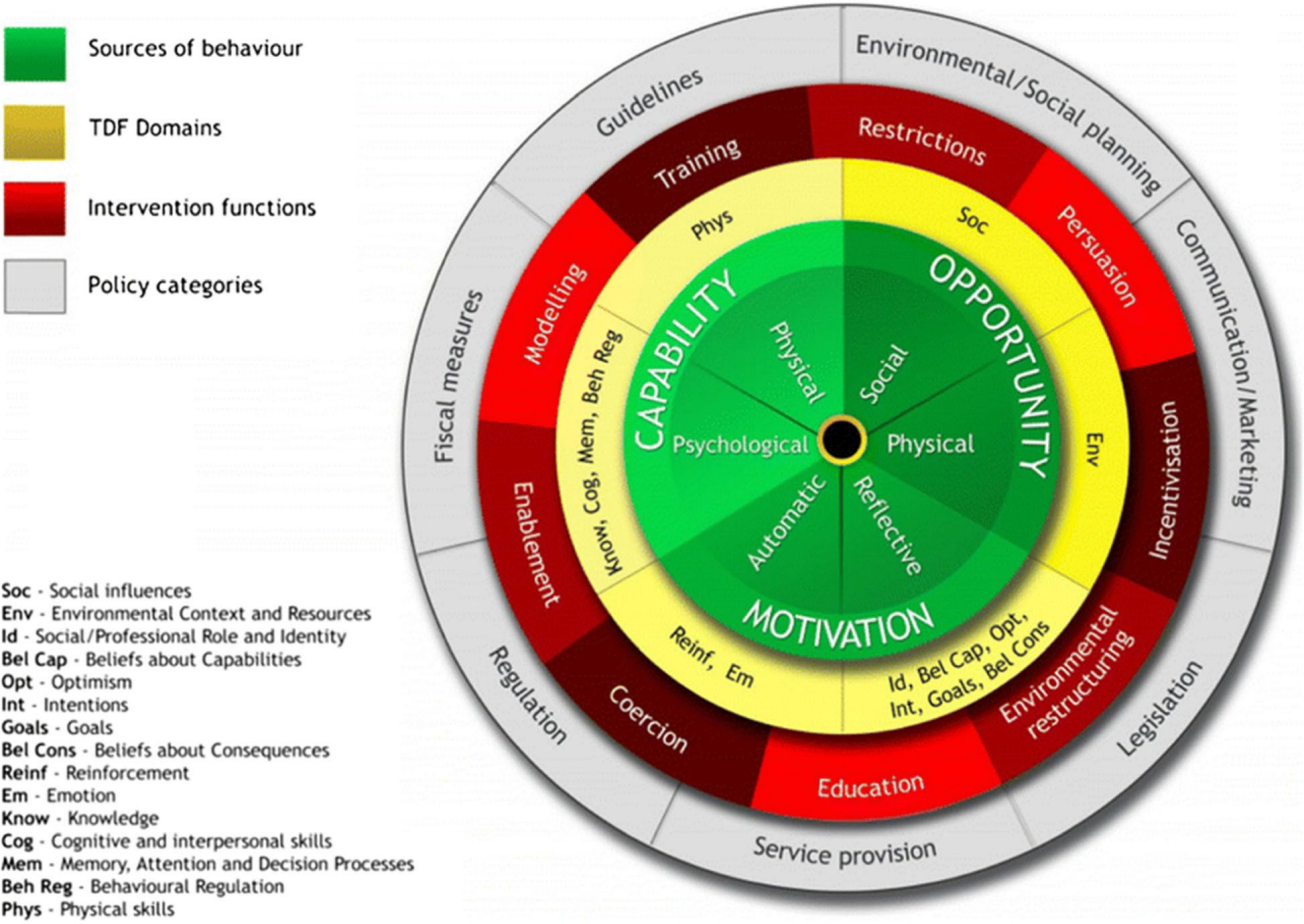
Behaviour Change Wheel, which highlights the COM-B model (green), TDF (yellow), intervention functions (red), and policy categories (grey) [33]

The BCW is a systematic tool that helps researchers transition from the behavioural diagnosis of a problem to designing and evaluating interventions to facilitate behaviour change (see Fig. 1). The BCW was developed from a broad range of nineteen multidisciplinary frameworks [33] and builds upon the MRC guidance. It offers a practical guide on how to develop theory- and evidence-based interventions [28]. The first step of intervention design, when applying the BCW, involves using the Capability, Opportunity, Motivation, Behaviour (COM-­B) model and the complementary Theoretical Domains Framework (TDF) to frame the behavioural diagnosis [29]. The COM-B model describes three different interacting categories that influence behaviour: (1) capability (physical and psychological capability), (2) opportunity (physical and social opportunity), and (3) motivation (reflective and automatic motivation). Further elucidation can be explored by using the Theoretical Domains Framework (TDF) [30, 32], which was added to the BCW to provide a more granular level of understanding of the three components of the COM-B model [34, 35]. Each domain of the TDF correlates to a COM-B component. The TDF is a meta-framework comprising 14 theoretical domains (such as ‘knowledge’, ‘skills’, ‘intentions’, and ‘social influences’) derived from 33 validated health and social psychology theories and over 128 behavioural change constructs designed to enable the systematic assessment of implementation issues to inform intervention design [30, 32]. It is a useful approach to understanding behaviours in diverse healthcare settings and was developed to support the implementation of new healthcare practices requiring behaviour change [34–38]. Using the COM-B model in combination with the TDF allows for a theory-informed analysis.

Once the behaviour has been analysed using the COM-B/TDF, the BCW can then be used to provide recommendations that can bring about change to the identified behaviours based on nine general types of intervention functions and seven policy categories. Following this, recommended strategies to support interventions can be achieved using the BCTTv1. This is a separate tool linked to the BCW consisting of theoretically informed or evidence-based behaviour change techniques to aid in the selection of interventions [29, 31, 39, 40]. The taxonomy includes 93 behaviour change techniques grouped within 16 categories, and several studies have applied the BCW and BCTTv1 to develop implementation interventions [27, 41, 42].

The purpose of this study is to develop a theory-driven understanding of the barriers and facilitators underpinning primary care physicians’ attitudes and capabilities to implementing SARS-CoV-2 point-of-care (POC) testing into routine clinical practice. This will enable the identification of strategies that could both encourage successful implementation of testing into routine practice and facilitate face-to-face consultations. To the authors’ knowledge, no research has explored the use of theory-driven studies to examine the challenges to adopting SARS-CoV-2 POC testing into routine practice and how these challenges can be addressed.

## Methods

### Design

**Fig. 2:**
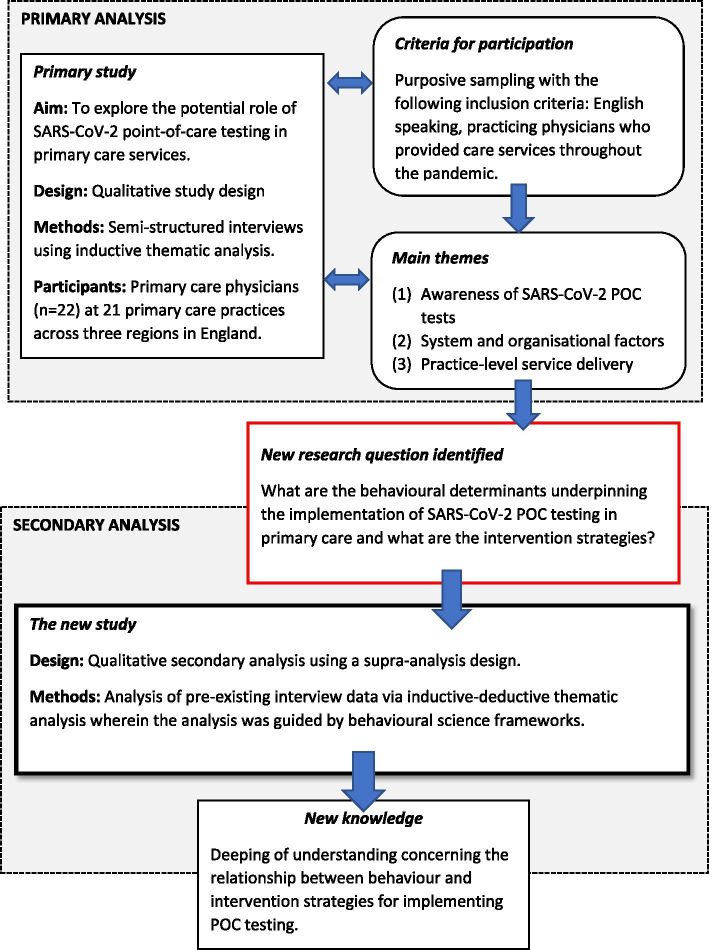
Transition from primary study to secondary analysis

This is a qualitative secondary supra-analysis that drew from pre-existing interview data collected from a primary qualitative study [43]. In the primary study, we sought to better understand the theoretical construction of where SARS-CoV-2 testing would best fit within the patient care pathway. For this, we used a grounded analytic approach [44]. The interviews did not originally set out to capture in-depth information that specifically explored the behavioural intentions and willingness of PCPs to adopt SARS-CoV-2 POC testing per se; however, there was a substantial discussion of these topics during the interviews. These described a range of complex behavioural phenomena affecting implementation. This enabled further analysis to allow us to gain a better understanding of the behavioural determinants that were likely to impact the uptake SARS-CoV-2 POC testing and to identify sustainable behaviour change strategies. Thus, the rationale for this secondary analysis was to illuminate these factors by re-examining our existing data and to generate new evidence by asking an empirical research question focused on the relationship between behaviour and intervention strategies (see Fig. 2) [45]. The Consolidated criteria for Reporting Qualitative Research (COREQ) was used to structure the reporting of the methods and results [46].

### Study participants and setting

Empirical data for the secondary analysis came from transcripts and notes obtained from 22 semi-structured interviews between September and November 2020. Study participants comprised a purposive sample of PCPs from 21 primary care practices across three regions (London, Thames Valley and South Midlands, North East and North Cumbria) in England. They were recruited with the assistance of three NIHR Local Clinical Research Networks (LCRNs). Participants were diverse with respect to years in practice, practice type, and geographical location (see Table 1). Interviews were conducted until we were confident that no new experiences or beliefs emerged [47, 48].

**Table 1:** Demographic features of participants and characteristics of study sites

Participant characteristics	Number
**Total number of participants**	22
**Sex**	
Male	12
Female	10
**Medical training**	
Average time post-qualification (years)	18
Range of qualification time [median] (years)	1–30 [19]
**Study site characteristics**	
**Region of practice**	
Thames Valley and South Midlands	9
London	4
North East and North Cumbria	8
**Number of patients registered to practice, mean**	14,522 (3600–40,000)
**Practice setting**	
Urban	7
Suburban	1
Rural	5
Mixed	8

### Source of data

All interviews were conducted online by four members (one male postdoctoral fellow, one female senior researcher, one female senior lecturer, and one male clinical scientist) of the research team who are experienced in qualitative methods. A semi-structured interview topic guide was used to prompt more detailed discussion led by the participants. The interview topic guide was developed iteratively by a multidisciplinary team including PCPs, evaluation methodologists, human factors specialists, and health economists. It was designed to conceptualise the patient care pathway during the pandemic to generate data that would inform a new pathway design to maximise the implementation of SARS-CoV-2 POC testing. The interview guides covered perceptions of point-of-care testing, local and national guidelines, and the perceived acceptability, feasibility, and challenges to implementation. The interviews, which lasted 45–60 min, were video-recorded using the Microsoft Teams software and then transcribed verbatim using the Otter.ai software. One participant did not consent to being recorded. Notes were recorded for that interview and included in our data analysis. All interview transcripts were checked against audio recordings. All recorded data were de-identified.

All participants who participated in the study provided informed verbal and written consent and were not compensated for participating in the study.

### Data analysis

**Fig. 3:**
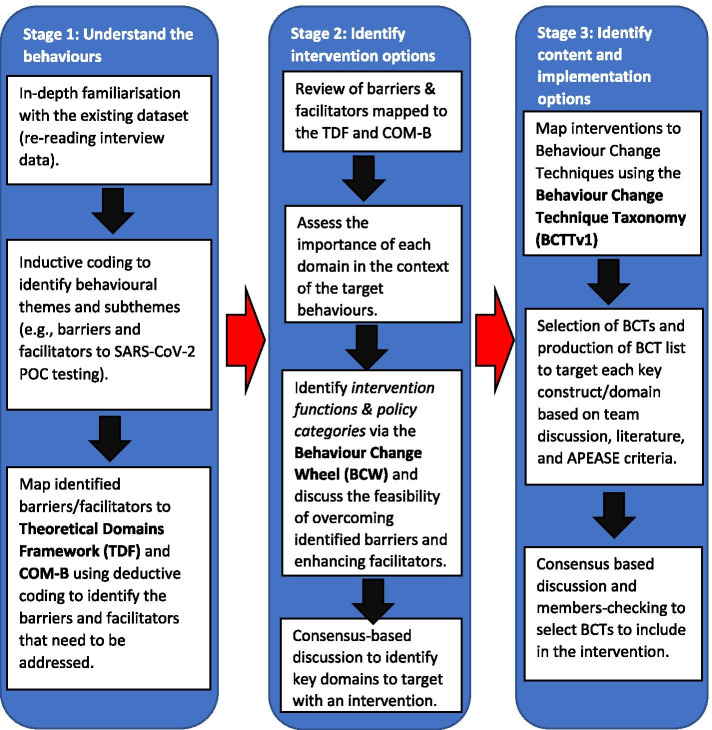
Overview of the data analysis process. This study comprises three stages: exploring PCPs’ perceptions of SARS-CoV-2 POC testing and using the TDF and COM-B to identify barriers to adoption (stage 1), identification of relevant behaviour change functions guided by the BCW to address key barriers (stage 2), and identification of potential targeted intervention strategies (stage 3)

The study followed the three stages recommended by the BCW: (1) understand the behaviours, (2) identify intervention options, and (3) identify content and implementation options [33]. Figure 3 provides an overview of the activities that took place within these three stages, and further details of these processes are provided in the text below.

#### Stage 1: Understand the behaviours

We used a combination of inductive and deductive approaches drawing from thematic analysis to understand PCPs’ attitudes and capabilities towards implementing POC testing into routine care practice [49]. An inductive approach was used to thematically analyse the data using the NVivo 1.3 software (QSR International). The first author (PK) developed the initial codebook and met with three team members (TH, AJA, YY) to refine the codebook and discuss potential themes and subthemes and define a consensus coding scheme (e.g. codes, definitions of codes, examples of quotes under each code). Thematic saturation was achieved when the research team judged that no new themes had emerged from the data [50]. The team proceeded with coding three more interviews and met again to discuss any new themes, resolve uncertainties, and examine for any convergence and divergence. Following this, all researchers coded the remaining interviews and met on a weekly basis to regularly check for consensus on coding. Discrepancies were solved through discussions until a consensus was reached with reference to the coding manual.

A deductive approach was used to match themes to the appropriate ‘domains’ within the TDF and COM-B to identify the attitudes and capabilities that could be targeted as potential levers of behaviour change [51–53]. During this stage, the first author (PK) re-read the data within the codes, allocated the themes to the appropriate TDF domains relevant for behaviour, and generated ‘belief statements’ across the domains that reflected the core beliefs expressed by the codes [30, 33]. To increase reliability of the assignment of theme-relevant TDF domain, a second coder (TH) independently mapped the themes to TDF domains until we were confident that there was agreement discussion to produce a ‘behavioural diagnosis’ (barriers and facilitators) for implementing POC testing into primary care. Discrepancies were resolved through discussion. A third researcher (AJA) checked the codes and their relevance to each TDF domain. All belief statements were verified by each analyst and adjusted for consensus. Any situations where a theme was mapped to one or more domains was discussed with a third team member (AJA) to reach consensus. The key domains considered likely to influence adoption were identified via three-pronged process. COM-B constructs and TDF domains that were considered of high importance based on the frequency of beliefs across the 21 study participants, presence of conflicting beliefs in the domain, and perceived strength of the belief that is believed to directly impact uptake (determined by consensus among the research team) [30].

#### Step 2: Identify intervention options

We identified intervention functions likely to elicit change in the TDF domains and COM-B components. Two members of the study team (PK, TH) used an iterative process to select and identify the most appropriate BCW intervention functions (education, persuasion, incentivisation, training, restriction, environmental restructuring, and enablement). Following this, we applied the intervention mapping matrix within the BCW framework which links COM-B and TDF domains to the intervention functions. Our discussions and decision-making were informed by the relevant literature, preferences outlined by participants in the interviews, and previous SARS-CoV-2 POC studies conducted by the authors of this study [54–59]. In addition, we applied the APEASE criteria (Affordability, Practicability, Effectiveness/cost-effectiveness, Acceptability, Side-effects/safety, Equity) to assess the appropriateness of each intervention function [29].

We proceeded with our behavioural analysis to identify and specify what internal and external conditions and actions needed to change to address each target behaviour identified in the empiric data. This involved two members of the team (PK, TH) identifying the theoretical constructs from the TDF and COM-B that needed to change for each specific behaviour to occur and most appropriate mode of delivery of each technique. These were then discussed with a third team member (AJA) for consensus. We relied on the experience of the research team which included clinicians, diagnostics specialists, and social scientists, together with feedback from other clinical colleagues to identify potential intervention options, to inform this process. In addition to these steps, we identified the relevant policy categories within the BCW to support the delivery of these interventions functions on a wider scale and evaluated against the APEASE criteria.

#### Step 3: Identify content and implementation options

Behaviour change techniques paired the intervention types identified via the BCW (based on the COM-B/TDF) with taxonomies in the BCTTv1 [29, 39]. Two members of the team (PK, TH) developed specific intervention strategies using information gathered from the qualitative interviews, literature on implementation strategies focused on changing professional practices in primary care [60–62], and our understanding of the context of what would be feasible to implement by consulting with clinician colleagues. We used the APEASE criteria to guide context-based decisions on the selection of appropriate intervention content [29]. This was further refined through member checking with colleagues (including primary and secondary care physicians) with clinical knowledge in diagnostics to verify the intervention strategies based on their perspectives on what could work based on the context of the study [63].

## Results

### Stage 1: Understand the behaviours

The analysis identified nine core themes thought to underpin PCPs’ attitudes and capabilities to implement SARS-CoV-2 POC testing. These included the following: (1) limited knowledge of the SARS-CoV-2 POC testing landscape, (2) scepticism about the insufficient evidence, (3) professional education and training, (4) PCPs would adopt POC tests if prescribed by authorities, (5) financial incentives, (6) limited workload capacity, (7) information sharing across practices, (8) society will view primary care as an alternative to community testing centres, and (9) perception of risk. We mapped these themes to eight out of the 14 TDF domains that were relevant to our study objective. The TDF domains included in our study were (1) knowledge, (2) behavioural regulation, (3) reinforcement, (4) skills, (5) environmental context and resources, (6) social influence, (7) professional role and identity, and (8) belief about consequences. Six of the other TDF domains were not included as they were determined by consensus discussion among the research team to have the least directly impact on SARS-CoV-2 POC testing uptake. Results are presented according to each of the core themes and relevant COM-B construct and TDF domain(s) with illustrative quotes in the main body of the paper. Table 2 provides an overview of how the themes, belief statements, and frequency were mapped to the COM-B constructs and TDF domains.

**Table 2:** Determinants to SARS-COV-2 POC test implementation: COM-B constructs and TDF domains identified and the corresponding key themes, frequency, and belief statements

COM-B constructs	TDF domains	Themes	Belief statements	No. of interviews discussing the theme (*n* = 22)
Psychological capability	Knowledge	1. Limited knowledge of the SARS-CoV-2 POC testing landscape	I am/am not familiar with POC tests and how they work.	20
		2. Scepticism about the insufficient evidence	I am/am not confident about the current evidence base.	15
Psychological capability	Behavioural regulation	3. PCPs would adopt POC tests if prescribed by authorities	I would/would not implement testing if asked to do so by local/regional/national authorities.	12
Physical capability	Skills	4. Professional education and training	I do/do not need training support to learn how to operate the tests safely and consistently.	18
Physical opportunity	Environmental context and resources	5. Limited workload capacity	I do/do not have time and resources to perform extra tasks.	18
Social opportunity	Social influences	6. Information sharing across practices	I am influenced/not influenced by the opinions of my colleagues and information shared on social media platforms.	12
Automatic motivation	Reinforcement	7. Financial incentives	I would/would not perform testing if I am paid to do it	19
Reflective motivation	Professional role and identity	8. Society will view primary care as an alternative to community testing centres	I am/am not worried that healthy members of the public will view us a testing facility.	18
Reflective motivation	Beliefs about consequences	9. Perception of assurance/risk	I will/will not feel safer about face-to-face interactions with patients.	21

### Mapping of themes with the COM-B constructs and TDF domains

#### Theme 1: Limited knowledge of the SARS-CoV-2 POC testing landscape (COM-B construct—psychological capability; TDF domain—knowledge)

PCPs had limited knowledge of the SARS-CoV-2 POC testing landscape, which mapped onto the COM-B component of psychological capability and the TDF knowledge domain. PCPs’ limited knowledge acted as a barrier as they were unable to identify the advantages or disadvantages of implementing POC tests into practice. These deficits in knowledge were largely attributed to the lack of available information surrounding POC testing, which negatively affected their willingness to adopt the tests.

I think there is a huge gap in knowledge around what point-of-care, antigen tests look like, how they work, the level of confidence we can have in the results and we’re hearing that even reading the results is variable. (GP 21)

#### Theme 2: Scepticism about the insufficient evidence (COM-B construct—psychological capability; TDF domain—knowledge)

The theme of scepticism about the insufficient evidence mapped to the COM-B component of psychological capability and the TDF knowledge domain. There were perceived doubts pertaining to the quality of evidence available concerning the POC tests that PCPs were somewhat familiar with. Their lack of confidence in the accuracy of tests was linked to the mixed body of evidence pertaining to the clinical efficacy and utility of POC tests.

It seems that most of the devices seem to be on based on a lateral flow model and I am not aware of any that have sort of received proof that they are valid and can be used as a decision-making tool in clinical practice. But as I say, I’ve not sort of looked into detail about what there is more broadly out there. (GP 08)

#### Theme 3: PCPs would adopt POC tests if prescribed by authorities (COM-B construct—psychological capability; TDF domain—behavioural regulation)

The theme of PCPs would adopt POC tests if prescribed by authorities maps to the COM-B construct of psychological capability and TDF domain of behavioural regulation. PCPs would integrate testing into practice if it was recommended via official guidelines and recommended by authoritative bodies.

If it was recommended by Public Health England or NICE, I think we would follow the guidelines. And the problem is that they are just changing so quickly, we have to rely on you know, the sources we’ve got available. So yeah, so if I think Public Health England said to us this test is a good test. You’re all using it, and then we’d have to trust it. (GP 02)

Another elaborated that they are obligated to follow guidance issued by their clinical commissioning groups.

General practices operate under the guidance from the local CCG and obviously the local CCG get advice from the NHS England in terms of what how we respond, and how we deal with things really. So, you would say the system level of how we operate is always based on the instruction there. (GP 11)

#### Theme 4: Professional education and training (COM-B construct—physical capability; TDF domain—skills)

In terms of professional education and training, factors related to physical capability (which mapped to the TDF Skills domain) included PCPs’ need for some support in terms of resources to prepare them to operate the tests efficiently. Although PCPs had some experience with providing service for other respiratory conditions requiring sample collection, there was a general consensus that training support would be beneficial and increase their confidence in testing.

All the people that work in the practice can take blood and do swabs, and quite a lot of us do respiratory stuff, spirometry and other breathing things. With simple training, we should be able to manage a point of care test that is simple, and it’s making sure it can be done repeatedly and accurately. (GP 16)

PCPs mostly referred to the need for healthcare assistants (HCAs) to receive training and take on the role as the main operators of the test.

I think it’d have to be a health care assistant specifically trained up to do that… it’s a skill that needs to be learned, but it’s quite a simple one. You need someone who’s focused on just that one problem. (GP 15)

#### Theme 5: Limited workload capacity (COM-B construct—physical opportunity; TDF domain—environmental context and resources)

The theme of limited workload capacity maps to the COM-B construct of physical opportunity and the TDF Environmental context and resources domain. PCPs expressed concerns that testing would add to existing work pressures. For PCPs, existing work would need to be alleviated, or compromises would have to be made to create capacity for them to provide SARS-CoV-2 testing services.

If we’re adding something new in… say there’s no new money, which too often isn’t, something else has to be taken away. It’s just not feasible to carry on doing everything and add in an extra thing. (GP 04)

For many, there were concerns about the feasibility of embedding POC testing into their practice without extra resources being made available to accommodate the anticipated changes of testing patients.

Adding point-of-care testing for COVID positive patients to our surgery, without adding staff and space…it won’t work. (GP 14)

#### Theme 6: Information sharing across practices (COM-B construct—social opportunity; TDF domain—social influences)

Information sharing across practices mapped to the COM-B construct of social opportunity and the social influences domain of the TDF domain. Participants discussed how information sharing across practices shaped their perception of POC tests, and some were wary of POC tests because of concerns expressed by colleagues.

But I think the general feeling I have, and I think most of my colleagues in the practice have is a lot of concern about that are they validated, and things like that, and our feeling, probably, broadly speaking, would be that it’s widely talked about by the government, but that would seem to be a political exercise. (GP 08)

PCPs also mentioned that information was regularly shared across platforms such as WhatsApp and Facebook. Generally, the types of information were not scientific articles and in most cases were linked to news media reports.

In terms of diagnostics, people have talked about it, but I’ve not really seen any kind of evidence-based information in those groups [social network platforms] yet about if there is one available for rapid testing. I mean, people have talked about that, posted articles which have been in the media. (GP 10)

#### Theme 7: Financial incentives (COM-B construct—automatic motivation; TDF domain—incentivisation)

Financial incentives map to the COM-B construct of automatic motivation and the TDF domain of reinforcement. Physicians reported that they would be willing to integrate testing into practice if they received financial incentives to cover the costs of the devices and employee time to perform the tests.

If you provide the machines, and you provide the consumables, and you pay for our time, we will do it. (GP 01)

#### Theme 8: Society will view primary care as an alternative to community testing centres (COM-B construct—reflective motivation; TDF domain—professional role and identity)

This theme maps to the COM-B construct of reflective motivation and the TDF domain of professional role and identity. Most interviewees perceived that the responsibility for administering POC testing should not primarily fall within the remit of primary care. There were concerns that the their professional identity would change in the eyes of the public if society began to view primary care as an alternative to community testing centres.

There’s a risk that we will start to get an increased demand of having a doing testing on people who are on have would fit in that category of mild symptoms and not needing a face-to-face appointment and that obviously has resource implications in terms of time and staff and staff costs from salaries. (GP 08)

Another explained that they believed patients would start viewing primary care sites as an attractive and convenient option when compared to existing testing options.

From a patient’s perspective, not surprisingly, that is very attractive. So, it doesn’t take a genius to work out that if you as a patient can get a near patient test for COVID, that’s going to be a very attractive commodity for patients. (GP 05).

#### Theme 9: Perception of assurance/risk (COM-B construct—reflective motivation; TDF domain—beliefs about consequence)

The theme perception of assurance/risk maps to the COM-B construct of reflective motivation and the TDF beliefs about the consequence domain. Factors related to these constructs/domains encompassed perceptions around the role of POC testing on increasing or decreasing the risk of contagion. PCPs would feel more confident about the benefits of implementing POC testing and providing more face-to-face appointments if the devices assisted them in ruling in and ruling out potentially infectious individuals.

It will make us more confident in face-to-face consultations. We’ve got a huge population with respiratory illness, especially COPD. I think these are the patients who kind of have missed out on getting seen, because any respiratory symptom they have an exacerbation, we really are relying on our clinical acumen and a kind of basic saturation maximum. Because we tend not to bring them in. So, these are the kind of patients especially with respiratory symptoms, who would benefit from a rapid testing, because then we can actually see them, or the patients who have weak symptoms who we don’t know if they have got COVID or not. (GP 10)

However, several also expressed concerns about the risk of occupational exposure. POC testing would equally place the practice staff at a higher risk of getting infected and losing manpower. For many, losing a staff member could threaten the sustainability of their practice.

One of the key vulnerabilities in this is the sustainability of the general practice service. You know, what we want to do is make sure that we don’t lose people, we don’t have to self-isolate... So, we’re losing manpower, and therefore productivity and sustainability. (GP 21)

### Stage 2: Identify intervention options

As outlined in the ‘Methods’ section (step 3), seven intervention functions from the BCW were considered useful and appropriate for addressing the identified barriers/enablers. These were ‘education’, ‘persuasion’, ‘training’, ‘enablement’, ‘incentivisation’, ‘environmental restructuring’, and ‘restriction’. The most common were ‘persuasion’ and ‘education’, which largely addressed the influence of knowledge and the role of information sharing. The remaining two intervention functions (i.e. ‘modelling’ and ‘coercion’) were excluded because they were either non-modifiable contextual factors considered not significant in determining PCPs’ attitudes and capability to adopt POC testing or did not meet the APEASE criteria and consensus-based group discussions. Coercion was not considered practicable, acceptable, or equitable as this was inconsistent with the overall requirements raised by PCPs. We excluded modelling as it was not considered relevant in the context of PCPs’ priorities raised in the data and through discussions with the wider research team. Following the recommendations of the BCW, we used the intervention function matrix to determine the most appropriate intervention functions for each component of the COM-B model (see Table 3). The seven intervention functions were mapped to the following six policy categories listed in the BCW guide: (1) communication/marketing—for example, the distribution of evidence-based information to generate awareness and reduce knowledge gaps around SARS-CoV-2 POC testing; (2) regulation—authoritative bodies prescribing the use of POC testing; (3) guidelines—such as producing and disseminating guidelines that are clear and concise; (4) service provision—provide training and course material; (5) fiscal measures—allocation of funding to compensate for the increased workload; and (6) environmental and social planning—providing infection prevention control (IPC) supplies to reduce the risk of contagion. Legislation was excluded for not meeting the APEASE criteria. The linkages between intervention functions and policy categories are provided in Table 4.

**Table 3:**
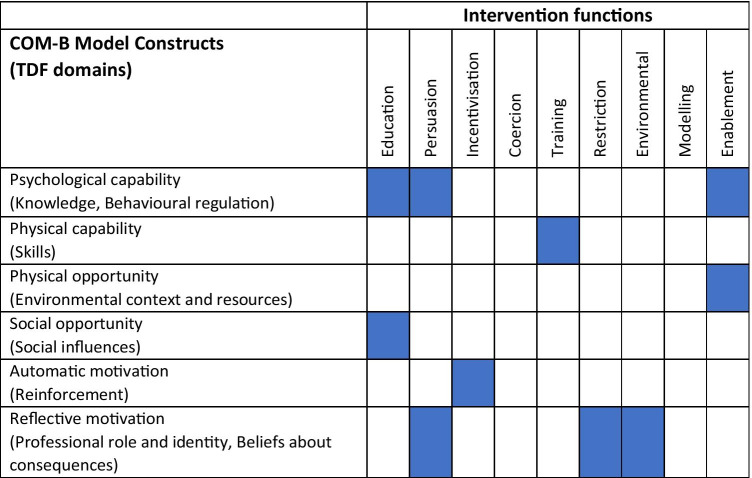
COM-B intervention function matrix: this table represents a matrix of barriers that were identified and the potential interventions to overcome them. The matrix is colour coded, and all blue-coloured areas represent where the COM-B/TDF aligns with the intervention functions

**Table 4:**
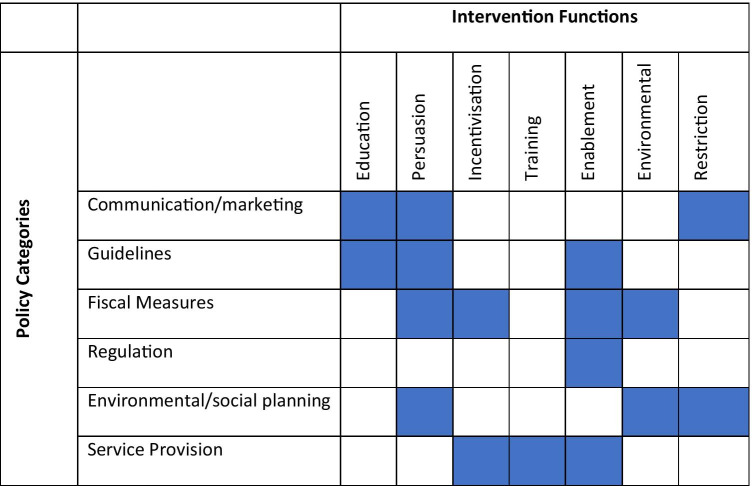
Linkage between intervention functions and policy categories. The blue-coloured areas represent the policy categories that can help support the delivery of the intervention functions

### Stage 3: Identify content and implementation options

Identification of behaviour change techniques was achieved by using the 93-item BCT taxonomy to identify 18 specific behaviour change techniques and map them to the intervention function that we considered would be relevant in future intervention. Examples of intervention functions for ‘training’ was mapped to the BCT technique ‘instructions on how to perform the behaviour’ to target staff’s need for training support in learning how to use POC tests. The most common techniques used were ‘information about social and environmental consequences’ (e.g. provide evidence-based information to cultivate confidence in the quality of POC tests). As described in the ‘Methods’ section, intervention strategies were evaluated against the APEASE criteria and further developed and refined through discussion with a group of physician colleagues who were part of our member checking team and were knowledgeable in care pathways analysis and evaluation of POC testing. The final mapping and linkage of the relevant themes, COM-B/TDF constructs, and intervention types, behaviour change techniques, and implementation strategies can be found in Table 5.

**Table 5:** Suggested interventions and descriptions using the behaviour change technique taxonomy (BCTTv1)

Themes	COM-B construct (TDF domain)	Intervention function(s)	Grouping and behaviour change techniques	Description of intervention strategies
Limited knowledge of the SARS-CoV-2 POC testing landscape	Psychological capability (knowledge)	Education, persuasion	Natural consequences - Information about social and environmental consequences Comparison of outcomes - Credible source	Distribute concise information with references from recognisable peer-reviewed journals summarising advantages and drawbacks of specific POC tests.
Scepticism about the insufficient evidence	Psychological capability (knowledge)	Education, persuasion	Natural consequences - Information about social and environmental consequences Comparison of outcomes - Credible source	Provide evidence-based information to cultivate confidence in the quality of POC tests.
PCPs would adopt POC tests if prescribed by authorities	Psychological capability (behavioural regulation)	Enablement	Goals and planning - Action planning goal (outcome)	Plan and prepare guidelines that physicians can better adhere to.
Professional education and training	Physical capability (skills)	Training	Shaping knowledge - Instructions on how to perform the behaviour Feedback and monitoring - Feedback on behaviour	Deliver specialised team training courses with supervision to ensure quality control of use. Ensure consistency in use. Tailor courses for healthcare assistants. Provide supervision and feedback to ensure proper device use.
Limited workload capacity	Physical opportunity (environmental context and resources)	Enablement	Reward and threat - Reward (outcome) - Non-specific reward Goals and planning - Problem solving Natural consequences - Information about social and environmental consequences	Provision of funding resources to increase staffing. Reduce or redistribute workload. Government funding needs to be allocated to primary care practices to increase staffing numbers.
Information sharing across practices	Social opportunity (social influences)	Education	Natural consequences - Information about social and environmental consequences Comparison of behaviour - Information about others’ approval Comparison of outcomes - Credible source	Increase PCP knowledgebase through the provision of evidence-based information. Equip PCPs with information to assess the quality of information shared across social network groups.
Financial incentives	Automatic motivation (incentivisation)	Incentivisation	Reward and threat - Material incentive Goal and planning - Behavioural contract	Contractual agreements between primary care practices and the authorities to provide payment to primary care practices to run the tests.
Society will view primary care as an alternative to community testing centres	Reflective motivation (professional role and identity)	Restriction, persuasion	Associations - Prompts/cues Natural consequences - Information about social and environmental consequences	Public health messaging to prevent the general public from identifying primary care practices as testing sites. Restrict access to testing only for individual’s requirement care.
Perception of assurance/risk	Automatic motivation (beliefs about consequences)	Restriction, environmental restructuring, persuasion	Antecedents - Avoidance/reducing exposure to cues for the behaviour Natural consequences - Information about health consequences Reward and threat - Reward (outcome) - Non-specific reward	Equip primary care practices with adequate supplies for infection prevention and control (IPC). Provide policies that will financially compensate primary care practice staff for the time they have to self-isolate.

## Discussion

This is, to our knowledge, the first study that utilised an implementation science framework to identify the barriers and specify the key intervention components to guide the implementation of SARS-CoV-2 POC testing into primary care. In doing so, this study addresses a clear evidence-practice gap at the intersection of implementation science and diagnostics. By using the BCW framework, our study unpacked a broad range of factors underlying PCPs’ attitudes and capability to implement POC testing and identified strategies that would help facilitate its integration into routine clinical practice. We discuss this in the following paragraphs.

First, knowledge gaps in SARS-CoV-2 POC testing negatively affected PCPs’ attitudes when it came to implementing testing into practice. Knowledge gaps can foster uncertainty [64, 65], as studies examining physicians’ perceptions of new medical devices found that perceived uncertainty of device effectiveness and lack of real-world evidence influenced physicians’ willingness to adopt the device [66, 67]. We also found that PCPs used passive information-seeking behaviour and incidental exposure to inform their knowledge of POC tests as opposed to purposefully seeking information on their own accord from sources they viewed as credible [68, 69]. This may be the outcome of several temporal challenges constraining PCPs from being able to allocate time to expand their knowledge around POC tests because of exhaustion, stress, and higher workload [70–72].

Considering this, educational interventions were identified as the means to address these challenges as information from credible sources can reduce uncertainties and effectively persuade and change attitudes with regards to the acceptance of new innovations [73–77]. However, the time pressures PCPs face in allocating resources to gather and process information suggest that providing credible sources may not be sufficient [78]. A balance needs to be struck in terms of the quantity and quality of information delivered to PCPs as providing too much information can also lead to information overload [79]. Educational interventions have to take into account the temporal factors underpinning PCPs’ availability to keep abreast with a constant flow of new and evolving information whilst managing their already demanding workload. This underscores the need for educational interventions to be designed in a way to deliver clear and concise information that will not noticeably interfere with existing workloads [80–82].

Second, PCPs expressed the need for professional training and education to ensure that they are equipped with the skills necessary to deliver the tests efficiently and confidently. This is consistent with studies which found that education and training can change the staff’s attitudes and improve clinical practice [83, 84]. Educational interventions could be delivered in the form of continuing medical education (CME) programmes, which PCPs are already accustomed to participating in to ensure that they provide optimal care based on the latest medical evidence [85–88]. Given PCPs’ demanding workload, internet-delivered CME activities and other digital learning resources may be able to help accommodate PCPs’ busy schedules by providing them a means to access professional training remotely and asynchronously at a time that is convenient for them [89, 90].

Alternatively, there was a broad consensus that healthcare assistants (HCAs) would be the best fit to operate the tests. HCAs are already accustomed to taking on responsibilities that remove excessive burdens from PCPs and nurses [91–93], and they may be willing to accept the new role within a practice as a result of normative influences [94]. Educational interventions will require the development of a standardised national training course to expand the role of HCAs to ensure that they acquire the appropriate skills and supervision to administer the tests [95, 96]. Nevertheless, it remains for further research to examine how testing responsibilities assigned to HCAs will have implications on job satisfaction levels and retention of staff given that they already feel underpaid and overworked [97, 98].

Third, PCPs would be willing to implement SARS-CoV-2 POC testing if it were issued as part of the guidelines prescribed by authoritative bodies. Despite this, the process of introducing new guidelines poses some challenges as PCPs are already burdened with adopting a plethora of guidelines sent to them from various organisations on a regular basis and at an unprecedented rate because of the pandemic [7, 99]. Thus, the dissemination of guidelines may not always result in practice changes despite physicians’ willingness to adopt them [100, 101]. Studies examining found that adherence to guidelines is highly dependent on the complexity of the guidelines and the frequency that they were updated [102]. In addition, several smaller practices may struggle to adopt guidelines as they would have to process large volumes of information with fewer staff in comparison with larger practices [103]. On this account, adherence will require that PCPs are provided with guidelines that are well-written and concise, offer summaries, and are clear about the changes proposed if they are to work within the boundaries of PCPs resource constraints [104–106].

Fourth, PCPs anticipated that implementing POC tests would threaten the sustainability of primary care if funding was not made available to accommodate the changes. The primary care system is already in a vulnerable position as it struggles with staff shortages and the challenges of physician recruitment and retention [107]. Failure to alleviate the intensity of PCPs’ workload would only add to the current woes of primary care, where there is a desperate need for more resources to increase the size of the primary care workforce [108]. Intervention strategies to address this requires funding to support the hiring and training of new staff to take on roles that have traditionally fallen within the domain of PCPs. For instance, physician assistants (associates) can reduce some of PCPs’ clinical duties at an overall lower cost [109–111]. In addition to this, pay-for-performance schemes were also suggested as a means to motivate PCPs to perform testing [112, 113]. However, this strategy requires some caution as government remuneration schemes have been shown to have little effect on counterbalancing the increasingly growing workload strain on PCPs [114], with prior research also highlighting that pay-for-performance schemes can lead to the de-prioritisation of other care tasks to reach financial targets [115–117].

Fifth, PCPs mentioned that different forms of information-sharing between colleagues influenced their perceptions of POC tests. This suggests that PCPs’ attitudes are influenced by the type of information exchanged within their professional network [94]. Studies have shown that people tend to accept others’ opinions as valid information [118, 119]. Intervention strategies could focus on leveraging these knowledge sharing activities by supplying PCPs with a higher quality of information to share across these networks to enhance PCPs’ perceptions of the benefits and drawbacks of POC testing [120, 121]. This may have a positive knock-on effect by motivating other PCPs to also contribute to sharing high-quality peer-reviewed information concerning the clinical utility of SARS-CoV-2 POC tests [122, 123].

Sixth, PCPs were concerned that their identity would be compromised if society began viewing primary care as an alternative to community testing services. This would also exacerbate existing concerns about unnecessary workload, given the concerns of patients inappropriately using NHS healthcare services that are not justified by clinical need [124]. Intervention strategies in the form of public health messaging may address these worries and support PCPs maintain their professional identity as it could help establish boundaries as to who can make an appointment request and subsequently get tested. This may nudge members of the public to pursue primary care services solely for clinical reasons and not testing [125]. It could also protect PCPs’ professional identifies and mitigate worries of testing not justified by clinical need adding onto the challenges of existing workloads [126–128].

Finally, PCPs believed the benefits of POC tests were their ability to remove clinical uncertainties between respiratory illnesses. This would generate confidence in terms of PCPs engaging in more face-to-face consultations. Yet, PCPs equally expressed concerns that increasing contact with patients significantly increases the chance of illness transmission and the need to quarantine. The increased likelihood of exposure to SARS-CoV-2 is known to increase stress and anxiety [129, 130] and can be financially devastating to practices [131]. Intervention strategies to address this will require that adequate supplies of protective equipment are provided together with the structural support to enhance infection prevention control measures at the health facility level, and policies to financially support primary care practices in the event staff members have to self-isolate [132].

This study highlights the importance of using implementation science to understand the complexities of implementing SARS-CoV-2 POC testing into primary care practices. Using an implementation science framework ensured that we used a standardised language of constructs to theoretically diagnose implementation challenges and inform intervention developments grounded in the collective experiences and views of PCPs working in diverse regions during the pandemic. It demonstrates that using the BCW has several strengths as it enhances our understanding and generalisability of the barriers/enablers to implementing new diagnostic tools into primary care and identify what needs to change to facilitate the integration of SARS-CoV-2 testing into routine care practice.

### Contribution to implementation science research

Although the BCW framework is well established, the findings in this study are the first to demonstrate its applicability in relation to systematically investigating and developing strategies to support the implementation of POC testing into primary care practices. We believe that our application of the BCW adds to the literature by addressing a gap at the intersection of implementation science and diagnostics and provides a transparent approach that can be replicated by other studies seeking to explore how to embed POC testing into routine clinical practice.

In addition, this study also demonstrates that the benefits of using secondary qualitative analysis open an avenue for methodological expansion when used in connection with implementation science. Although the primary study was not originally designed with the use of implementation science frameworks in mind, the study still demonstrated that secondary qualitative analysis paired with the BCW can provide researchers with the opportunity to re-examine data, analyse new hypotheses, and inform different research questions to further deepen knowledge and reveal additional context around PCPs’ attitudes and capabilities to implementation. Such approaches can help implementation researchers efficiently save time and resources, especially when it comes to obtaining data from hard to recruit study participants.

### Limitations

A limitation to this work is that it was predominately based on the perspectives of PCPs and did not include a larger sample of other patient-facing professionals. The inclusion of the perspectives of other patient-facing professionals in future studies would be valuable. This study was also conducted across 21 primary care practices in three regions in England, and therefore, transferability may be limited. Another limitation is that data was originally collected between September 2020 and October prior to the second national lockdown in November 2020. It is possible that some participants may have changed their perspectives on testing and priorities. It is worthy to note that the multistep process of the BCW was a lengthy and time-consuming process. Application of the TDF resulted in some limitations as some codes were difficult to assign to one specific domain. Participants in this study were presented with hypothetical scenarios and with no experience of using a SARS-CoV-2 test. Their opinion might change in the event they gain such experience. Finally, whilst the findings provide us with insight into the implementation barriers/enablers and suggested potential intervention strategies, it is not possible to comment on how successful our findings would be when translated into real-world settings. Further refinement of the interventions proposed in this study will need to undergo feasibility and pilot testing.

## Conclusions

In this study, we identified barriers and enablers in the uptake of POC tests for SARS-CoV-2 in the primary care pathway. Our findings suggest that there are a broad number of interdependent barriers and enablers at the clinician, organisational, and system levels. Interventions to address the barriers should involve improving PCPs’ knowledgebase of high-quality studies demonstrating the clinical utility of POC tests, to incentivise testing, introduce policies to help embed testing into practice, and provide resources to primary care practices to meet the anticipated demands of testing. The findings of this study can be used to help inform policymakers and decision-makers improve testing dissemination strategies.

## Data Availability

All relevant data are within the manuscript and its supporting information files. The datasets used and/or analysed during the current study are available from the corresponding author on reasonable request.

## Abbreviations

BCTTv1: Behaviour Change Technique Taxonomy Version 1
CCG: Clinical commissioning group
COM-B: Capability, Opportunity, Motivation, Behaviour
COVID-19: Coronavirus disease 2019
PCP: Primary care practitioner
HCA: Healthcare assistant
LCRN: Local Clinical Research Network
LMC: Local Medical Committees
MRC: Medical Research Council
NIHR: National Institute of Health Research
POC: Point-of-care
TDF: Theoretical Domains Framework
QOF: Quality and Outcomes Framework
SARS-CoV-2: Severe acute respiratory syndrome coronavirus 2
